# Oil frying enhances flavor and sensory quality of salted dried mackerel: a comparative study of heating methods

**DOI:** 10.3389/fnut.2025.1640819

**Published:** 2025-10-27

**Authors:** Jing Li, Caiyan Jiang, Yan Liu, Xiaoqing Miao, Pengfei Jiang

**Affiliations:** ^1^SKL of Marine Food Processing & Safety Control, National Engineering Research Center of Seafood, School of Food Science and Technology, Dalian Polytechnic University, Dalian, China; ^2^School of Information Science and Engineering, Dalian Polytechnic University, Dalian, China

**Keywords:** salted and dried Spanish mackerel, thermal processing method, volatile flavor substances, free amino acids, sensory quality

## Abstract

This research primarily examines the effects of three thermal processes–air frying, water steaming, and oil frying–on the volatile and non-volatile flavor compounds of salted Spanish mackerel dried meat (with a salt concentration of 6% and drying durations of 4 and 6 days). The results indicated that 45 volatile flavor compounds were discovered using GC-IMS, with the oil frying group exhibiting the highest concentration of desirable volatiles. The e-tongue findings indicated that the savory flavor was the most prominent across all sample groups. According to LC-MS measurement of free amino acid content, the concentration of fresh flavor amino acids was most pronounced in the oil frying group. The oil frying group also earned the highest overall rating in the sensory evaluation. This research provides a theoretical framework for the development of prepared meals with salted and dried Spanish mackerel.

## 1 Introduction

The popular Spanish mackerel (*Scomberomorus niphonius*) possesses firmer, fattier flesh, fewer spines, a powerful, sweet, and salty flavor, and is nutrient-dense. It is predominantly located in China’s East China Sea, Bohai Sea, and Yellow Sea, with substantial consumer markets in South Korea, Japan, and China (1). Moreover, the consumption of mackerel is embedded in the cultural traditions of China’s coastal areas. Curing is a conventional method for preserving fresh fish that relies on natural air drying and salt as the sole seasoning (2). Post-curing, air frying aquatic items provide benefits like as a chewy texture, altered structure and organization, and decreased processing costs. Furthermore, curing can extend the shelf life of Spanish mackerel while also modifying its flavor (3). Two essential elements that influence the quality of cured fish are the duration of curing and the concentration levels. If the concentration is excessively low, the salt’s penetration and diffusion cannot achieve equilibrium, rendering the final product devoid of a salty flavor (4). Excessive marinade concentration or extended marinating duration can exacerbate the oxidation of proteins and lipids, resulting in adverse impacts on the fish’s texture, color, and flavor (5).

A primary quality aspect affecting customer decision-making is food flavor (6). The flavor precursors in raw ingredients and thermal processing methods predominantly determine the taste of meat products (7). The composition of flavor compounds generated in raw materials by various thermal processing methods will vary significantly, resulting in distinct meat flavors, even when the flavor precursor composition in the raw meat remains unchanged (8). The heat transfer medium employed in diverse thermal processing methods differs considerably, leading to changes in the composition, physical and chemical properties, and sensory quality of food raw materials, which are perceptible to the naked eye to differing extents. Prevalent thermal processing methods encompass baking, steaming, boiling, microwave heating, and air frying. Air frying is a novel technique that, while not involving traditional frying, can yet impart a flavor like to that of fried foods (9). Research by Uran and Gokoglu (10) indicates that roasting is the optimal method for cooking anchovies, since it maximally preserves the fish’s nutrition and results in a firm, flavorful product. Roasting at 180 °C for 20 min yields optimal nutritional composition and qualitative attributes of anchovies. Fan et al. (11) investigated the impact of several heat processing procedures on the physicochemical properties and flavor components of *Procambarus clarkii*. Wei et al. (12) employed sensory analysis to assess the steamed and fresh yellow croaker filets. The steamed meat exhibited a robust, fatty aroma that enhanced the overall odor acceptability of the large yellow croaker. Presently, the majority of research on the flavor of Spanish mackerel primarily examines the influence of different fish sections or the processing parameters of a singular thermal processing technique on flavor development (13). Nevertheless, there exists a paucity of research regarding the impact of various thermal processing techniques on the flavor and volatile component profile of dried salted Spanish mackerel meat.

Gas chromatography-ion mobility spectroscopy (GC-IMS) is presently the predominant method employed in food flavor research. It surpasses other detection systems in data monitoring, screening, and comprehensive information retrieval (14). The advantages of GC-IMS encompass affordability, ease of operation, rapid response time, elevated sensitivity, and the absence of necessary sample preparation (15).

The capability of liquid chromatography-mass spectrometry (LC-MS) to quantify endogenous or exogenous small molecule compounds–predominantly byproducts or oligomers of chemical reactions in biological systems–facilitates a thorough examination of the metabolic activity and state of cells and organisms (16). Utilizing LC-MS technology to quantify the taste compounds in each sample group and assess the free amino acid content of dried salted Spanish mackerel meat under different heating conditions enabled the expression of variations in the taste substance content of the meat across these conditions.

The primary focus of this investigation was dried salted mackerel meat cured for 4 or 6 days with a 6% salt concentration. Additional objectives encompassed identifying the three prevalent thermal processing techniques for ripening dried salted Spanish mackerel meat: air frying, water steaming, and oil frying; evaluating the flavor profile of the dried salted Spanish mackerel meat utilizing GC-IMS; and assessing the free amino acid composition of the dried salted Spanish mackerel meat under diverse heating methods through LC-MS, electronic tongue analysis, and integration with sensory evaluation. The findings provide a theoretical basis for the quality of dried salted Spanish mackerel meat under different heat processing methods.

## 2 Materials and methods

### 2.1 Materials and instruments

Changhai County, Dalian City, Liaoning Province, China, provided fresh Spanish mackerel (*Scomber scombrus*); China Salt Industry Company Limited supplied the salt; Yihaijiali Food Marketing Co., Ltd., supplied the vegetable oil; Beijing Baoshidi Co., Ltd., supplied chromatographic-grade methanol; Aladdin Biochemical Science and Technology Co., Ltd., supplied acetonitrile; and the remaining reagents were of analytical purity.

HD9651 Air Fryer, Philips, The Netherlands; T18 Homogenizer, IKA, Germany; CR22N High-Speed Refrigerated Centrifuge, Hitachi, Japan; TS-5000Z Electronic Tongue, Insent, Japan; Discovery HR-1 FlavourSpec^®^ Flavor Analyzer, G.A.S, Germany; Vanquish Ultra High-Performance Liquid Chromatography, Thermo, USA; Q Exactive HFX High Resolution Mass Spectrometer, Thermo, USA.

### 2.2 Sample preparation

The merchant pre-treated the sample on the market (length 35–45 cm, weight 350–450 g), incising it along the spine to excise its internal organs and cleansing it of blood stains and other residues. The Spanish mackerel was subsequently salted at a concentration of 6% (material-liquid ratio of 1:10) and dehydrated in a cold, well-ventilated environment in late October 2022. This yielded the requisite salted dried Spanish mackerel sample for the experiment. Samples designated for 4 and 6 days with a 6% salt concentration were selected for diverse thermal processing methods. The desiccated Spanish mackerel was selected from the dorsal muscle, cut into uniform dimensions (5 cm × 4 cm × 0.5 cm), and thereafter desalinated for 3 h, with water changes implemented every 90 min.

Thermal processing methods encompass water steaming, wherein fish pieces are arranged in a stainless-steel steamer drawer at 100 °C for 10 min; oil frying, which entails submerging fish pieces in vegetable oil heated to 210 °C, with the oil volume maintained at approximately 10% of the total mass of the fish; and air frying, where fish pieces are uniformly placed in an air fryer and cooked for 8 min at 180 °C. The experimental groups were labeled S64 and S66 for water steaming, F64 and F66 for oil frying, and A64 and A66 for air frying. The fish segments were flipped midway through the duration of each thermal processing technique.

### 2.3 Determination of volatile flavor substances

After undergoing several thermal processing processes, the dried Spanish mackerel samples were shredded, accurately weighed at 2.0 g, placed in a 20.0 mL headspace injection vial, incubated for 20 min at 40 °C, and subsequently analyzed for volatile flavor components using the Floorspace^®^ Flavor Analyzer. Alluding to the methodology of Huang et al. (17) with some adjustments. The instrument settings are as follows: Headspace incubation temperature: 40 °C; incubation duration: 20 min; rotating speed: 279 × g; injection needle temperature: 85 °C; injection volume: 500.0 μL, non-split mode; carrier gas: high-purity N_2_ (purity ≥ 99.99%); cleaning duration: 0.5 min.

Gas chromatography parameters: column temperature: 60 °C; operating time: 25 min; carrier gas: high-purity N_2_ (purity ≥ 99.99%); flow rate started at 2.0 mL/min and was maintained for 10 min before increasing to 150 mL/min in 15 min.

Ion mobility spectroscopy parameters: MXT-5 column (15 m in length, 0.53 mm in inner diameter, and 1 μm in thickness); column temperature: 60 °C; drift gas (N_2_, purity ≥ 99.99%); flow rate: 150 mL/min; IMS temperature: 45 °C; analysis duration: 20 min.

### 2.4 Quantification of non-volatile flavor substances

#### 2.4.1 Taste determination by electronic tongue

Following multiple heat processing procedures, take 10 g of desiccated Spanish mackerel samples, introduce 100 mL of deionized water, and homogenize using a homogenizer. Subsequently, transfer the homogenate to a centrifuge and centrifuge for 10 min at 715 × *g*. Following centrifugation, extract the supernatant, filter it, and transfer it into an electronic tongue sample cup for analysis and uploading. Each sample was measured four times, and the results of the final three measurements were documented and analyzed.

#### 2.4.2 LC-MS analysis (determination of free amino acids)

The principal processing technique involved selecting an appropriate sample quantity, meticulously disaggregating it, and subsequently grinding it in a grinder until it attained a useful powder form. Subsequent to introducing the sample into the centrifuge tube, incorporate 1 milliliter (0.1 M) of hydrochloric acid and mix vigorously using a vortex mixer. Centrifuge the sample at 4 °C for 10 min at 134 × *g* following an hour of equilibration at room temperature. Subsequently, dilute the supernatant at the appropriate intervals. Introduce 10 μmol/mL of the sample into the vial, followed by the addition of 20 μmol/mL of the AccQ-Tag reagent and 70 μmol/mL of the AccQ-Tag Ultra Borate buffer.

In positive ion mode, a 0.1% aqueous solution of formic acid served as mobile phase A, while a 0.1% acetonitrile solution of formic acid constituted mobile phase B. The column temperature was 55 °C, the injection volume was 1 μL, and the flow rate was 0.5 mL/min. The samples were maintained in an autosampler at 4 °C during the evaluation period. To alleviate the effects of the instrument’s variable detection signal, the samples were subjected to ongoing analysis in a randomized order.

#### 2.4.3 Taste active value (TAV)

Taste active value can be used to quantify how much of a flavor ingredient contributes to the sample’s overall flavor (18). TAV > 1 indicates that the substance has a substantial impact on flavor and that the value and contribution are positively connected; TAV < 1 indicates that the substance has no discernible impact on taste. Here is the formula:


T⁢A⁢V=C/T

C is the content of the taste substance, ug/mL; T is the threshold of the taste substance, mg/100 mL.

#### 2.4.4 Sensory evaluation

The dried salted mackerel meat was sliced into uniformly sized pieces (5 cm × 4 cm × 0.5 cm) and placed onto a dish after being air frying, water steaming, and oil frying. Sixteen professionally trained sensory evaluation experts (between 23 and 26 years of age), eight of whom were male and the other eight of whom were female, were asked to conduct a sensory evaluation on the various dried salted mackerel meat products that had undergone heat processing. Each of the following index scores ranged from 0 to 10, with 0 representing color and luster (0 points for black-brown fish pieces and 10 for bright and shiny fish pieces), 0 for fishy smell, halo smell, and 10 for the distinct aroma of salted fish, 0 for taste (0 for salty saltiness or lighter taste is poor and 10 for moderate salinity, strong aftertaste, and dense meat), and 0 for chewing (0 for soft or hard meat, no chewing). Chewiness (0 points for firm or soft meat that doesn’t require chewing, 10 points for compact flesh that does), overall assessment (1 point for subpar, 10 points for superb).

Note: The sensory experiment was approved by Dalian Polytechnic University and all participants participated voluntarily and signed a right to information.

### 2.5 Statistical analysis

The collected experimental data were organized and analyses using Microsoft Office Excel. The consolidated data underwent the Duncan’s multiple-range test (*P* < 0.05) utilizing IBM SPSS Statistics 26 analytical software. The experimental results were presented as mean ± standard deviation (the experiments were repeated three times or more). Volatile flavor components in different hot-processed dried salted mackerel were analyzed using VOCal software. The chemicals were evaluated qualitatively utilizing the NIST database and the IMS database of the software. The Reporter and Gallery plug-ins were employed to generate the difference map and fingerprint of volatile flavor compounds, whereas Prism 10.0 software and Origin 2019 were used for plotting.

## 3 Discussion and analysis

### 3.1 GC-IMS analysis of various thermally processed dried salted Spanish mackerel’s meat

**FIGURE 1:**
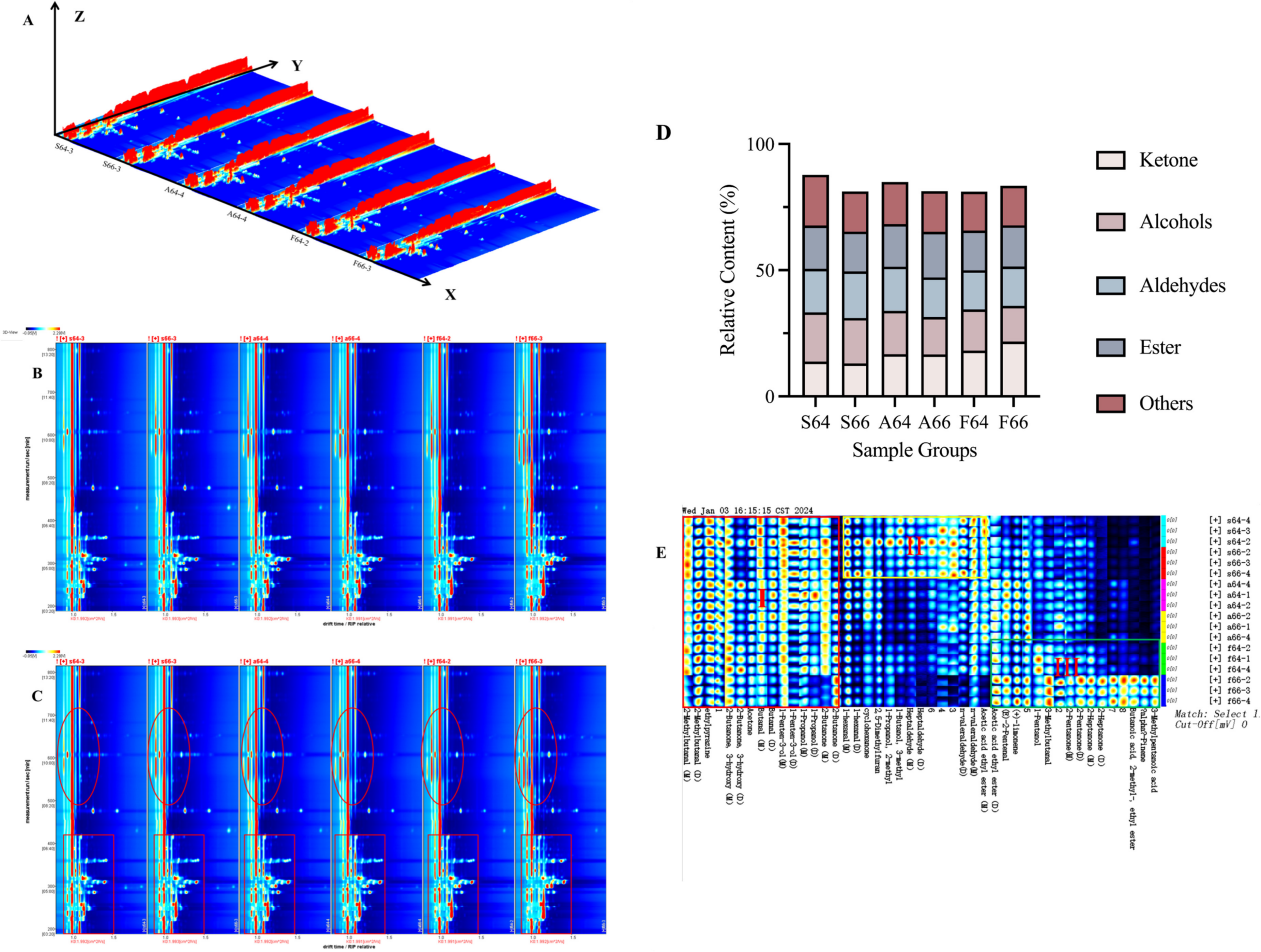
Volatile organic compounds in three different thermally processed dried salted Spanish mackerel meat. **(A)** GC-IMS 3D map; **(B)** GC-IMS 2D map; **(C)** Comparison of differences using the S64-3 group as a reference; **(D)** Classification ratio of volatile components in dried salted mackerel meat under different thermal processing modes; **(E)** GC-IMS profiles of dried salted mackerel meat under different thermal processing methods.

The comprehensive data on volatile flavor compounds in beef subjected to different heating procedures can be obtained from the GC-IMS spectra. Each peak in Figure 1A signifies a volatile flavor compound; a darker peak indicates a greater concentration of the chemical. The figure demonstrates that the volatile flavor compounds in different salted and heated dried mackerel foods possess analogous compositions, although vary in their concentrations. The group that steamed water demonstrated the highest quantity of flavoring chemicals, closely succeeded by the group that fried oil. Figures 1B, C illustrate that the migration time of volatile flavor compounds in the meat of dried salted mackerel predominantly falls within the range of 1.0–1.5, whilst the retention duration spans from 200 to 700 s. The oil frying group demonstrated the most pronounced signal of volatile flavor compounds throughout the retention time of 200–450 s, referred to as the rectangular zone. Likewise, the elliptical region, associated with the 500–700 s retention time, had the most pronounced signal of volatile flavor compounds.

### 3.2 Qualitative analysis of volatile flavor substances between different thermally processed dried salted Spanish mackerel meat

Qualitative and quantitative analysis of volatile flavor components was conducted by correlating with the GC-IMS database (refer to Table 1). Considering Figure 1C and Table 1, it is apparent that the dried salted mackerel meat, after undergoing various thermal processes, had a total of 45 volatile compounds: 12 aldehydes, 10 ketones, 7 alcohols, 3 esters, 5 miscellaneous substances, and 8 uncharacterized compounds. The total concentration of all volatile compounds in each sample was regarded as 100% to more effectively demonstrate the contribution of each volatile ingredient to the overall flavor of salted dried mackerel following thermal processing. The proportion of each volatile chemical relative to the total concentration was subsequently computed and represented in percentage stacking diagrams, as illustrated in Figure 1D. The heated salted dried mackerel exhibits elevated levels of two principal flavor components, aldehydes and ketones, as illustrated in the figure.

**TABLE 1:** Volatile flavor compounds identified in different thermally processed dried salted Spanish mackerel meat.

Count	Compound	Odor type	CAS	Formula	MW	RI	RT [sec]	Dt [a.u.]
1	Cyclohexanone	Mint, cool	C108941	C6H10O	98.1	1256.6	736.19	1.15536
2	2-butanone-3-hydroxy (M)	Butter	C513860	C4H8O2	88.1	1296.1	798.508	1.05784
3	2-butanone 3-hydroxy (D)	–	C513860	C4H8O2	88.1	1296.1	798.508	1.05784
4	1-pentanol	Balsamic, fruit, green, pungent, yeast	C71410	C5H12O	88.1	1263.9	747.599	1.25699
5	1-butanol-3-methyl	Fresh, fruity, floral	C123513	C5H12O	88.1	1222.1	684.877	1.2481
6	2-heptanone (M)	Blue cheese, fruit, green, nut, spice	C110430	C7H14O	114.2	1196.7	649.276	1.26431
7	2-heptanone (D)	–	C110430	C7H14O	114.2	1197.3	650.123	1.63352
8	Heptaldehyde (M)	Citrus, fat, green, nut	C111717	C7H14O	114.2	1199.9	653.72	1.33143
9	Heptaldehyde (D)	–	C111717	C7H14O	114.2	1200.8	654.895	1.69418
10	(+)-limonene	Citrus, mint	C10H16	C10H16	136.2	1175.7	608.524	1.21915
11	(E)-2-pentenal	Stimulating	C1576870	C5H8O	84.1	1118	501.479	1.0957
12	1-hexanal (M)	Apple, fat, fresh, green, oil	C66251	C6H12O	100.2	1103.2	477.353	1.26195
13	1-hexanal (D)	–	C66251	C6H12O	100.2	1103.3	477.391	1.56143
14	1-propanol, 2-methyl	Apple, bitter, cocoa, wine	C78831	C4H10O	74.1	1109.7	487.73	1.17323
15	1-propanol (M)	Alcohol, candy, pungent	C71238	C3H8O	60.1	1056.1	418.021	1.11201
16	1-propanol (D)	–	C71238	C3H8O	60.1	1055.8	417.661	1.25055
17	2-pentanone (M)	Fruit, pungent	C107879	C5H10O	86.1	1002.2	360.623	1.12265
18	2-pentanone (D)	–	C107879	C5H10O	86.1	1001.4	359.901	1.3728
19	N-valeraldehyde (M)	Almond, bitter, malt, oil, pungent	C110623	C5H10O	86.1	1004.8	363.243	1.18203
20	N-valeraldehyde (D)	–	C110623	C5H10O	86.1	1003.8	362.29	1.42471
21	Alpha-pinene	Cedarwood, pine, sharp	C10H16	C10H16	136.2	1015.3	373.872	1.21583
22	2,5-dimethylfuran	Savory	C625865	C6H8O	96.1	970.5	337.087	1.04677
23	3-methylbutanal	Chocolate, malty	C590863	C5H10O	86.1	915	300.994	1.19509
24	Butanal (M)	Banana, green, pungent	C123728	C4H8O	72.1	892	287.173	1.12221
25	Butanal (D)	–	C123728	C4H8O	72.1	891.3	286.747	1.28367
26	Acetone	Pungent	C67641	C3H6O	58.1	839.5	257.978	1.11697
27	2-methylbutanal (M)	Almond, cocoa, fermented, hazelnut, malt	C96173	C5H10O	86.1	925.1	307.213	1.15973
28	2-methylbutanal (D)	–	C96173	C5H10O	86.1	925.9	307.717	1.39955
29	Acetic acid ethyl ester (M)	Aromatic, brandy, grape	C141786	C4H8O2	88.1	921.3	304.864	1.09239
30	Acetic acid ethyl ester (D)	–	C141786	C4H8O2	88.1	925	307.184	1.32737
31	2-butanone (M)	Fragrant, fruit, pleasant	C78933	C4H8O	72.1	916.6	301.955	1.06026
32	2-butanone (D)	–	C78933	C4H8O	72.1	917.1	302.289	1.24743
33	Butanoic acid, 2- methyl-, ethyl ester	Apple, ester, green apple, kiwi, strawberry	C7452791	C7H14O2	130.2	1029.3	388.49	1.23675
34	3-methylpentanoic acid	Fetid	C105431	C6H12O2	116.2	948.3	322.096	1.27067
35	Ethylpyrazine	Burnt, green, iron scorch, must, peanut butter, roasted, rum, wood	C13925003	C6H8N2	108.1	945.4	320.225	1.13384
36	1-penten-3-ol	Butter, fish, green, oxidized, wet earth	C616251	C5H10O	86.1	1176.2	609.665	0.94754
37	1-penten-3-ol (D)	–	C616251	C5H10O	86.1	1176.5	610.173	1.34507
38	1	–	Unidentified	*	0	831.2	253.646	1.20909
39	2	–	Unidentified	*	0	832	254.113	1.28465
40	3	–	Unidentified	*	0	912.6	299.51	1.86891
41	4	–	Unidentified	*	0	908.5	296.976	1.67315
42	5	–	Unidentified	*	0	1030.4	389.584	1.03425
43	6	–	Unidentified	*	0	1175.5	608.155	1.58726
44	7	–	Unidentified	*	0	1144.3	547.777	1.1568
45	8	–	Unidentified	*	0	1144.9	548.984	1.2413

*Filling in the molecular formula table for unidentified substances.

The fundamental aldehydes, heptanal, hexanal, pentanal, and butanal, may arise from the thermally induced oxidative cleavage of certain unsaturated fatty acids. Hexanal predominantly imparts a green flavor at appropriate concentrations; however, excessive amounts can result in a rotten and disagreeable taste (19). Hexanal is a result of the degradation of unsaturated fatty acids by peroxide (20). Table 1 indicates that the dried salted mackerel meat from the water steaming group exhibited significantly elevated concentrations of hexanal, glutaraldehyde, and 2-methylbutanal compared to the dried mackerel meat from the air frying and oil frying groups. Furthermore, the food had a distinctive scent attributable to ketones, aldehydes, and alkanes, which are byproducts of lipid thermal degradation. Although individual aldehydes generally possess a powerful odor, trace aldehydes can augment the food’s fragrance, rendering it more subdued and viscous. The flavor of the dried salted mackerel from the water steaming group was inferior to that of the air frying and oil frying groups.

The key factors contributing to ketone creation were fresh, fruity, and clear fragrances, resulting from the oxidative degradation of fatty acids and the oxidative production of alcohols. In comparison to the other two groups, the oil frying group exhibited a markedly greater volume of ketone peaks in the dried salted mackerel meat. The unsaturated fatty acids in the oil may have expedited lipid oxidation in the meat during frying, while the elevated frying temperature further intensified this process, resulting in an increased ketone concentration in the oil frying group (21).

The primary alcohols are 1-penten-3-ol and n-pentanol. Alcohols has a higher odor threshold compared to other substances and do not substantially influence the flavor of dried salted mackerel. Alcohols are generally generated via the oxidative degradation of lipids. In this process, the carbon chain of fatty acids is cleaved, releasing free radicals that interact with the hydroxyl group to produce alcohols. This procedure can impart a refreshing, invigorating fragrance to aquatic objects (22). The n-amyl alcohol concentration in the oil frying group was markedly greater than in the other two groups. This may be due to the oxidative degradation of linoleic acid in the dried salted mackerel during frying, resulting in a fatty aroma in the meat. The dried salted mackerel meat subjected to various thermal treatments exhibited an elevation in alcohol concentration.

Esters are the principal contributors to the flavor profile of dried salted mackerel and significantly augment its overall taste. They possess a delightful fruity fragrance and mitigate the undesirable odor resulting from the oxidation of fatty acids. Esters are recognized for their low olfactory detection threshold. The principal esters are ethyl 2-methylbutyrate and ethyl acetate, the latter exhibiting a distinct fruity flavor. Among the thermal processing processes, oil frying generated the highest quantity of esters. The oil frying group demonstrated the most significant lipid oxidation owing to the rapid heat transfer and minimal heat loss throughout the procedure. This resulted in an increased production of free fatty acids and, subsequently, the highest level of esterification in the oil frying group (23).

In total, five supplementary groups of chemicals, comprising furan, pyrazine, pentene, and pentatonic acid, were identified in the dried salted mackerel meat both before to and following thermal processing. The taste of dried salted mackerel is significantly improved by pyrazine compounds, which are consistently generated during thermal processing. Despite their aggregate proportion being modest, their thresholds are considerably low.

### 3.3 GC-IMS fingerprint analysis of different thermally processed dried salted Spanish mackerel meat

The amalgamation of Figure 1E and Table 1 reveals that the volatile flavor compounds present in the dried salted Spanish mackerel meat across the six groups were predominantly attributed to the Maillard reaction, esterification, oxidative degradation of lipoproteins, and their interactions. The discovered volatile flavor chemicals were predominantly aldehydes, ketones, alcohols, esters, and heterocyclic compounds. The flavor of meat products is mostly attributed to the oxidative degradation of lipids. Upon heating, unsaturated fatty acids generated from lipid hydrolysis can undergo oxidative degradation, yielding a diverse array of aldehydes, alcohols, and ketones, with aldehydes possessing a minimal threshold for markedly affecting flavor. Lipid oxidation leads to the oxidation of arachidonic acid, oleic acid, and linoleic acid, yielding unsaturated aldehydes and linear aldehydes such as heptanal, hexanal, and glutaraldehyde (24). Oxidative amino acid degradation generally yields branched-chain aldehydes such as 3-methylbutyraldehyde and 2-methylbutyraldehyde, primarily generated through the Strecker reaction that degrades lysine, isoleucine, and leucine. All six sample groups contained ethyl acetate and ethyl 2-methylbutyrate, which are byproducts of lipid and free fatty acid oxidation that frequently yield esters. The types and quantities of volatile flavor compounds produced differed based on the extent of the preceding reactions of the dried salted Spanish mackerel during the three thermal processing techniques.

### 3.4 PLS-DA and heat map analysis

**FIGURE 2:**
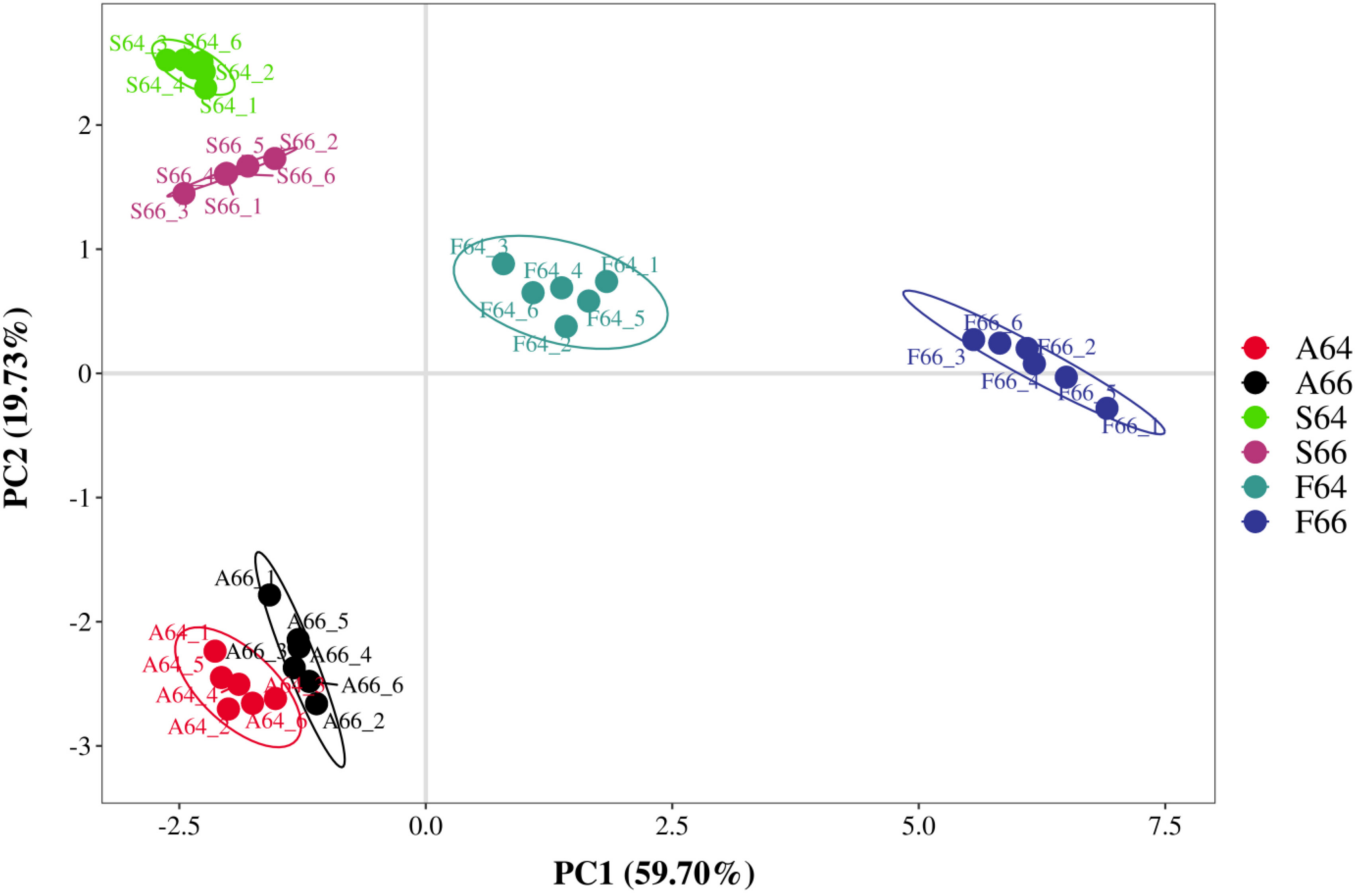
Partial least squares discriminant analysis (PLS-DA) analysis of volatile substances in different thermally processed dried salted Spanish mackerel meat.

The volatile flavor compounds of dried salted mackerel meat under various heating modes were analyzed using supervised Partial Least Squares Discriminant Analysis (PLS-DA) to investigate the key constituents of the characteristic flavor profile of salted mackerel dried meat across different heating conditions from the 37 volatile flavor compounds identified. Figure 2 illustrates that the flavor profile of three varieties of thermally-processed dried salted mackerel meat can be characterized by four primary components. The figure clearly demonstrates that the model can differentiate among the three types of heat-processed dried salted mackerel meat. The supplementary calculations of the model’s fit R^2^ and prediction Q^2^ yielded a cumulative fit R^2^(cum) = 0.916 and a cumulative prediction Q^2^(cum) = 0.889, indicating the model’s robust predictability and adaptability, as well as its capacity to differentiate among various types of thermally processed dried salted mackerel meat.

**FIGURE 3:**
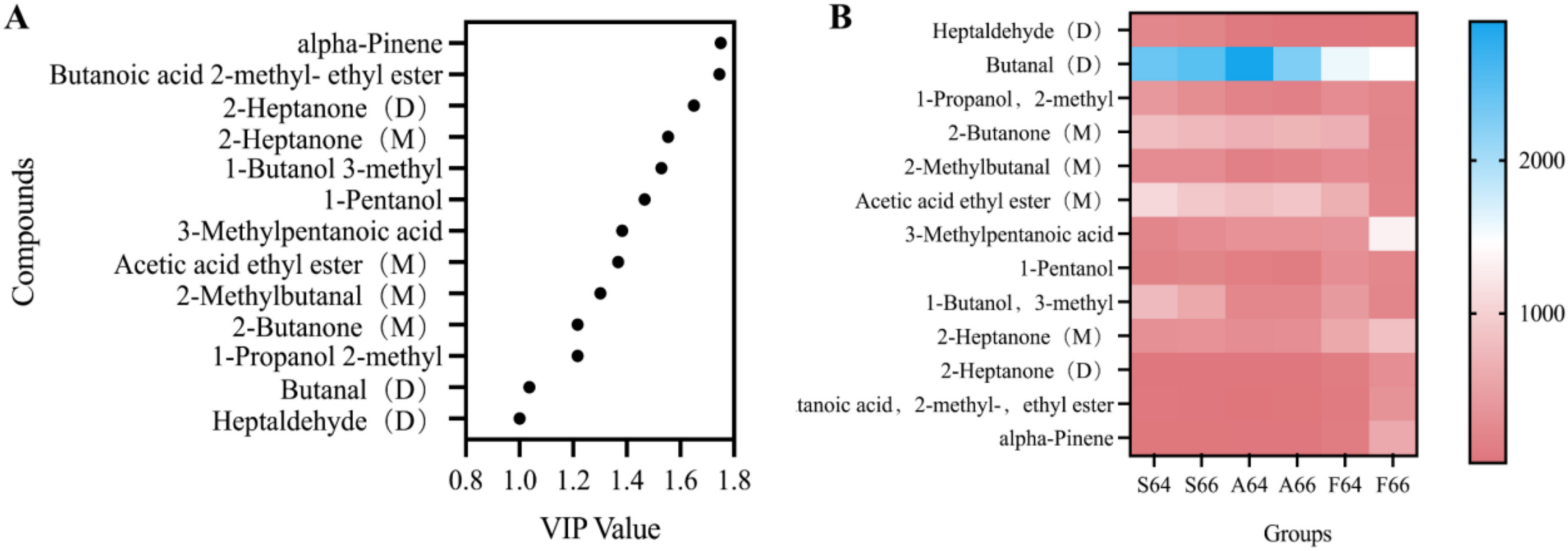
Variable importance projection (VIP) values **(A)** and heat maps **(B)** of some volatile substances in different thermally processed dried salted Spanish mackerel meat.

Variable importance projection (VIP), an essential output parameter of the PLS model, effectively and precisely indicates each variable’s contribution to the model’s capacity to differentiate the samples (25). When VIP > 1, it is recognized that this variable significantly contributes to the model’s discrimination, as illustrated in Figure 3A.

Figure 3A illustrates the 13 volatile flavor chemicals with a VIP greater than 1. The molecules exhibiting the highest VIP values are alpha-pinene (1.751), butanoic acid, 2-methyl-ethyl ester (1.746), 2-Heptanone (D) (1.650), and 2-Heptanone (M) (1.554). The most prominent volatile flavor chemicals were alpha-pinene, 2-methyl-ethyl ester (1.746), and 2-heptanone (D) (1.650). Higher VIP ratings signify that this volatile flavor component exhibits greater variability when subjected to different heating methods. The scent of dried salted Spanish mackerel jerky under different cooking conditions was mostly ascribed to alpha-pinene and butanoic acid, 2-methyl-ethyl ester, accompanied by notes of green apple and cedar, respectively.

Figure 3B distinctly illustrates the differences in volatile flavor components among the dried meat of salted mackerel subjected to various heat treatments. The oil frying group had markedly elevated levels of alpha-pinene, butanoic acid, 2-methyl-ethyl ester, 2-Heptanone (D), and 3-methylpentanoic compared to the other two groups. Consequently, these four volatile flavor compounds can be utilized to characterize the oil used for frying dried salted Spanish mackerel flesh. Butanal (D) was shown to have the maximum concentration exclusively in air-fried dried salted mackerel meat, indicating that this flavor compound can be utilized to characterize the air frying of dried salted mackerel meat. In the water-steamed dried salted mackerel, the concentration of acetic acid ethyl ester (M) was markedly greater than in the other two groups, indicating that this flavor compound can be utilized to characterize water-steamed dried salted mackerel. As a result, a distinct variation in flavor was seen among the dried salted mackerel meat produced by each of the three thermal processing methods.

### 3.5 Electronic tongue analysis

**FIGURE 4:**
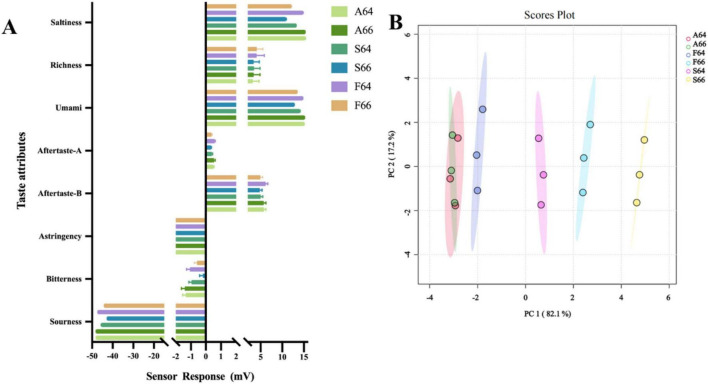
Electronic tongue **(A)** and PCA analysis **(B)** of dried meat of different thermally processed salted Spanish mackerel.

An electronic tongue system was employed to evaluate the flavor profile of dried salted Spanish mackerel meat subjected to different thermal processing techniques (26, 27). Figure 4A presents the response values for fresh taste, astringent aftertaste, and bitter aftertaste across the samples of each group subjected to different heating processes, arranged in the following order. The water steaming group exhibited the minimal presence of bitter and astringent aftertaste, however no significant difference was seen between the oil frying and air frying groups. This can be elucidated by the production of bitter chemicals in both frying methods. This can be attributed to the observation that the bitter chemicals generated during oil frying and air frying operations were less in the water steaming group. Figure 4A clearly indicates that the primary flavor qualities are salty and fresh. This occurs mainly because inorganic ions infiltrate the fish during the curing process, resulting in the degradation of proteins into tiny peptides and amino acids, as well as the deterioration of nucleotides (28). This is consistent with previous findings (29, 30). In contrast to the air frying and oil frying groups, the electronic tongue exhibited significantly lower response values for salty and fresh flavors in the water steaming group; this may be attributed to the substantial water vapor generated during the steaming process, which diluted the salty and fresh flavors in the dried salted mackerel. Furthermore, compared to the samples prepared by steaming and frying, the dried salted mackerel cooked in the air fryer demonstrated a heightened level of freshness, likely due to conduction heating facilitating the formation and accumulation of gustatory compounds. The group that prepared the mackerel in oil exhibited moderate levels of freshness and saltiness, likely due to the vegetable oil reducing the salinity and freshness of the dried salted mackerel.

Principal components analysis (PCA) was employed to elucidate the flavor characteristics of dried salted mackerel meat under different heating temperatures, as depicted in Figure 4B. PC1 and PC2, with contribution rates of 82.1% and 17.2% respectively, and a cumulative contribution rate of 99.3%, demonstrated significant responsiveness to the predominant data concerning the general attributes of dried salted mackerel, thereby enabling the characterization of the meat’s flavor profiles when subjected to various heating methods. All groups were separated from each other, save for the two that experienced oil and air frying. This indicated that although the fundamental attributes of the flavor presentation persisted, the electronic tongue could differentiate the taste characteristics of the dried salted mackerel meat after it underwent treatment with three distinct heating methods: oil frying, air frying, and water steaming.

### 3.6 LC-MS analysis of free amino acids

Liquid chromatography-mass spectrometry was utilized to elucidate the flavor-associated biochemical processes in dried salted mackerel meat subjected to different heating settings (31, 32). This approach efficiently separates and identifies metabolites in dried salted mackerel meat under diverse temperature conditions (33). Free amino acids are principally categorized into three groups: fresh, bitter, and sweet (34). These groups significantly influence the flavor profile of dried salted mackerel flesh. Table 2 illustrates that the air frying group exhibited the highest concentrations of phenylalanine, leucine, and valine, particularly in the A66 subgroup. Conversely, the water steaming group demonstrated elevated levels of arginine, serine, and L-Citrulline, most notably in the S64 subgroup. The oil frying group recorded the highest amounts of aspartic acid, glutamic acid, and 4-hydroxy-L-proline, especially in the F64 subgroup. Aspartic acid and glutamic acid are the primary contributors of umami flavor (35). The computerized tongue assessment of fresh flavor indicated that the oil frying group exhibited significantly higher values compared to the other two groups. Free amino acids, encompassing the bitter amino acids (leucine, isoleucine, and valine) and the sweet amino acids (serine and threonine), will interact with the e-tongue’s sensors, resulting in alterations in taste perception (36).

**TABLE 2:** Composition (mg/L) of free amino acids in different thermally processed dried salted Spanish mackerel meat.

Types of free amino acids	A64	A66	S64	S66	F64	F66
Histidine	173.65 ± 22.35a	250.92 ± 68.42a	273.86 ± 71.41a	231.62 ± 23.13a	243.59 ± 46.92a	169.43 ± 11.2a
4-hydroxy-L-proline	2.85 ± 0.31a	3.37 ± 0.96a	4.24 ± 1.32a	2.76 ± 0.11a	4.26 ± 0.96a	2.20 ± 0.17a
Arginine	14.12 ± 1.88c	41.11 ± 13.35a	35.93 ± 9.69ab	27.20 ± 1.35abc	30.83 ± 6.24abc	21.24 ± 0.33bc
Asparagine	0.06 ± 0.02a	0.15 ± 0.07a	0.06 ± 0.01a	0.07 ± 0.01a	0.09 ± 0.05a	0.13 ± 0.14a
Glutamine	3.94 ± 0.64b	8.60 ± 3.1ab	11.97 ± 3.01a	9.12 ± 1.28a	10.97 ± 1.08a	7.61 ± 0.06ab
Serine	11.49 ± 1.30b	25.21 ± 8.37a	21.54 ± 5.47ab	19.69 ± 1.82ab	19.52 ± 1.9ab	15.14 ± 0.05ab
Glycine	30.04 ± 3.28a	38.57 ± 7.70a	34.45 ± 4.48a	38.93 ± 0.95a	33.29 ± 0.05a	34.34 ± 0.07a
Aspartic acid	1.71 ± 0.09b	16.24 ± 5.88a	2.29 ± 0.52b	6.63 ± 0.98b	6.16 ± 0.13b	3.92 ± 0.19b
L-citrulline	2.36 ± 0.18ab	3.62 ± 1.26ab	3.93 ± 1.12a	3.74 ± 0.25ab	1.98 ± 0.26b	3.38 ± 0.02ab
Glutamic acid	22.89 ± 3.90b	47.89 ± 16.96a	27.20 ± 7.34ab	38.54 ± 5.21ab	34.70 ± 0.60ab	29.60 ± 0.14ab
Threonine	29.14 ± 5.32a	40.44 ± 13.37a	40.40 ± 10.82a	36.59 ± 2.93a	33.90 ± 3.07a	27.18 ± 0.22a
Alanine	68.85 ± 11.46a	102.35 ± 34.70a	101.25 ± 25.66a	102.13 ± 11.64a	89.51 ± 2.87a	77.12 ± 1.55a
Gamma-aminobutyric acid	0.31 ± 0.05a	0.39 ± 0.25a	0.43 ± 0.14a	0.32 ± 0.01a	0.31 ± 0.00a	0.28 ± 0.04a
Proline	20.28 ± 3.82b	45.40 ± 16.27a	25.51 ± 7.11b	32.38 ± 2.48ab	23.89 ± 2.30b	25.10 ± 0.85b
L-ornithine	9.30 ± 1.92a	12.22 ± 5.09a	10.08 ± 2.54a	15.36 ± 2.56a	8.52 ± 0.49a	11.31 ± 0.76a
D-2-aminobutyric	1.19 ± 0.24ab	1.76 ± 0.65a	0.87 ± 0.17b	1.92 ± 0.18a	0.82 ± 0.00b	1.24 ± 0.15ab
Lysine	90.67 ± 20.12b	227.65 ± 114.6ab	272.14 ± 80.95ab	178.27 ± 32.10ab	185.83 ± 22.76ab	118.58 ± 8.72ab
Cystine	0	0	0	0	0	0
Tyrosine	14.05 ± 1.80b	30.68 ± 7.26a	21.95 ± 6.23ab	20.53 ± 0.86ab	19.15 ± 4.90ab	17.64 ± 1.16b
Methionine	12.14 ± 2.14b	27.15 ± 8.27a	20.02 ± 5.46ab	19.52 ± 1.18ab	16.53 ± 3.25ab	16.22 ± 0.25ab
Valine	18.06 ± 3.13b	36.17 ± 12.35a	27.17 ± 7.58ab	30.85 ± 2.71ab	25.06 ± 3.14ab	24.82 ± 0.04ab
Isoleucine	12.52 ± 2.09b	29.02 ± 10.09a	20.86 ± 5.58ab	22.27 ± 1.82ab	17.30 ± 2.11ab	18.14 ± 0.19ab
Leucine	23.88 ± 4.04b	58.40 ± 20.15a	44.66 ± 12.07ab	42.95 ± 3.29ab	34.10 ± 4.06ab	34.67 ± 0.26ab
Phenylalanine	14.84 ± 1.38b	31.16 ± 7.54a	25.67 ± 6.87ab	19.64 ± 0.37ab	19.48 ± 4.78ab	17.28 ± 1.02b
Tryptophan	3.02 ± 0.18b	6.22 ± 1.39a	4.06 ± 1.06b	3.88 ± 0.02b	3.18 ± 0.76b	3.54 ± 0.18b

Results are represented as mean values ± standard deviation (*n* = 3). Different letters followed by the results in the same row indicate significant differences (*P* < 0.05) according to the Duncan’s multiple-range test.

**FIGURE 5:**
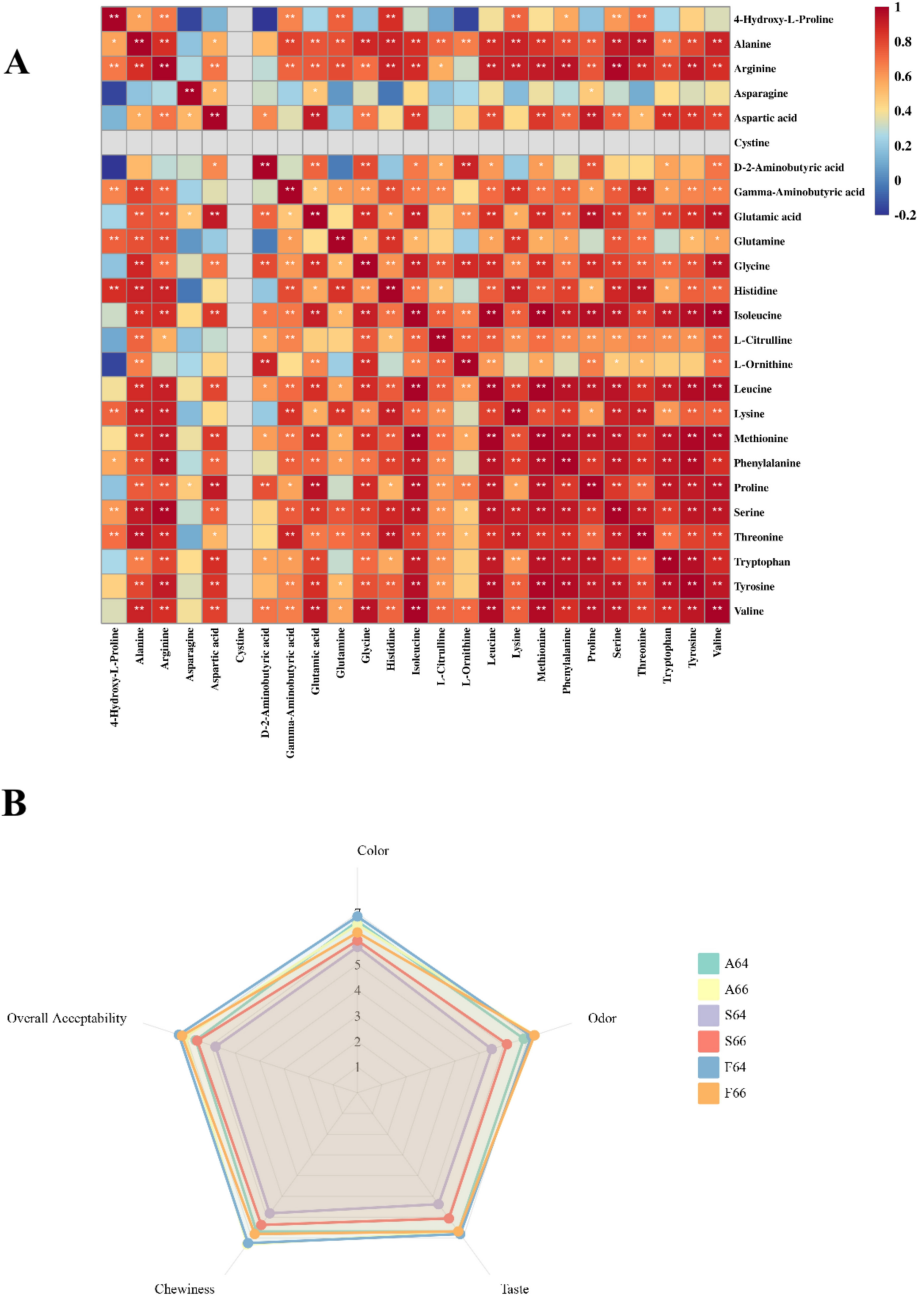
Correlation analysis between free amino acid **(A)** and sensory evaluation chart **(B)** in different thermally processed dried salted Spanish mackerel meat.

Subsequent analysis of the association between taste values and free amino acids indicated (Figure 5A) that L-ornithine had a positive correlation with sour, salty, and astringent flavors. Conversely, the water steaming group exhibited a markedly elevated content of L-ornithine compared to the other two groups, indicating that the dried salted Spanish mackerel meat in that group would possess an undesirable flavor. Research by Sun et al. (37) indicates that the bitter receptor has a greater affinity for the proline binding site, leading to an intense bitter taste. Table 2 indicates that the proline levels in the water steaming and air frying groups are markedly elevated compared to the oil frying group, whereas the aspartic acid and glutamic acid concentrations in the oil frying group are comparatively substantial. The results indicate that the flavor of the oil frying group surpasses that of the other two groups, and this is positively connected with the taste alteration identified by the electronic tongue.

Various amino acid types and amounts can impart sweet, sour, and bitter flavors to foods (38). Table 3 indicates that, after thermal processing, the concentration of glycine was significantly higher than that of the other free amino acids, and that the primary contributor to the sweet flavor was TAV above 1, corroborating the results of Jin et al. (39). Arginine is a bitter amino acid with a subtle sweetness that enhances food freshness and flavor complexity. Elevated levels of arginine in the fish enable it to synergize with swe anine, imparting a distinctive flavor to the dried salted mackerel. Proline is an amino acid utilized for sweetening food; it mitigates the flavor of undesirable substances, such as dried salty mackerel, while also imparting sweetness. The concentration of fresh and sweet amino acids was significantly greater than that of bitter amino acids after thermal treatment. The rationale may be that heating the desiccated salted Spanish mackerel will, firstly, induce hydrolysis of proteins and peptides, yielding fresh amino acids such as glycine; secondly, the liberated amino acids will participate in reactions such as decarboxylation and deamination, transforming these compounds into aldehydes, hydrocarbons, amines, etc., consequently diminishing the concentration of bitter amino acids. As stated by Yang et al. (40), cooked pufferfish meat possesses a more pronounced sweet flavor compared to raw pufferfish meat. This is also due to the higher concentrations of glutamic acid, aspartic acid, glycine, and alanine in cooked pufferfish meat.

**TABLE 3:** Taste active value (TAV) analysis of flavor-presenting amino acids in dried salted Spanish mackerel meat under different thermally processed.

Free amino acid	Form	Taste threshold (mg/100 mL)	A64	A66	S64	S66	F64	F66
Histidine	Bitter	200	86.825	125.46	136.93	115.81	121.795	84.715
Arginine	Bitter	1306.5	0.10807501	0.314657482	0.275009568	0.20818982	0.235973976	0.162571757
Lysine	Bitter	1169.6	0.775222298	1.946391929	2.326778386	1.524196306	1.588833789	1.013850889
Tyrosine	Bitter	72.44	1.939536168	4.235229155	3.030093871	2.834069575	2.64356709	2.435118719
Valine	Bitter	351.45	0.513871105	1.029164888	0.773082942	0.877792005	0.713045952	0.706217101
Isoleucine	Bitter	90	0.954268293	2.211890244	1.589939024	1.697408537	1.318597561	1.382621951
Leucine	Bitter	144.32	1.654656319	4.046563193	3.094512195	2.976025499	2.362804878	2.402300443
Phenylalanine	Bitter	743.4	0.199623352	0.419155233	0.345305354	0.264191552	0.262039279	0.232445521
Serine	Sweet	262.75	0.437297812	0.959467174	0.819790676	0.749381541	0.742911513	0.57621313
Glycine	Sweet	187.75	1.6	2.10758988	1.834886818	2.073501997	1.77310253	1.829027963
Threonine	Sweet	416.85	0.699052417	0.970133141	0.969173564	0.87777378	0.813290949	0.652033105
Alanine	Sweet	106.92	6.439393939	9.572577628	9.46969697	9.552001496	8.371679761	1.850065971
Proline	Sweet	287.75	0.704778454	1.577758471	0.886533449	1.125282363	0.830234579	0.87228497
Aspartic acid	Umami	53.24	0.321187077	3.050338092	0.430127724	1.245304282	1.157024793	0.736288505
Glutamic acid	Umami	16.18	14.14709518	29.59826947	16.81087763	23.81953028	21.44622991	18.29419036
Cystine	Odorless	24.22	0	0	0	0	0	0
Methionine	Odorless	74.6	1.627345845	3.639410188	2.683646113	2.616621984	2.215817694	2.174262735

The free amino acid thresholds in the TAV calculations are all referenced to George A (41).

### 3.7 Sensory evaluation

**FIGURE 6:**
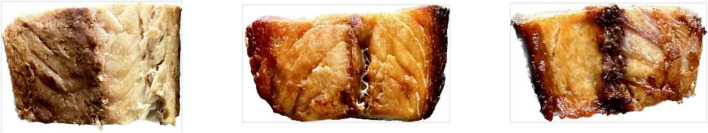
Finished products of dried salted mackerel with different thermal processes. From left to right are water steaming, air frying and oil frying.

Figure 6 illustrates the completed salted and dried Spanish mackerel subjected to several heat treatments. Analyses of the volatile flavor and taste components of dried salted mackerel meat, conducted using an electronic tongue, GC-IMS, and LC-MS with various heating procedures, revealed an exceptionally rich flavor. Therefore, a sensory assessment was performed on it. The dried salted mackerel exhibited a homogeneous color distribution and a natural yellow-brown hue after heat treatment (Figure 5B). The oil frying group demonstrated a markedly superior color score compared to the other groups, especially the F64 group, and exhibited a more pronounced yellow-brown hue. The unique umami and salty flavor of salted mackerel was perceptible through its aroma in each group. All dried mackerel, which had undergone salting and heat processing, was fresh, sweet, and salty, devoid of any bitterness. The oil frying group outperformed the air frying and water steaming groups in terms of chewiness and overall acceptability. The feel of the steaming water group was more enticing. The group subjected to oil frying obtained the highest evaluation scores, especially the F64 group.

## 4 Conclusion

Three distinct thermal processing methods were evaluated for their effects on volatile flavor components, concentration of taste compounds, and sensory quality of dried salted mackerel flesh. The results revealed that 45 distinct volatile flavor compounds were discovered by GC-IMS, with aldehydes and ketones being the predominant substances. Among the volatile flavor compounds with elevated concentrations were butanal (D), 3-methylpentanoic acid, butanoic acid, 2- methyl-, ethyl ester, and others. The most notable finding was that the oil frying group–specifically the F64 group–exhibited the highest concentration of volatile flavor compounds compared to the others. Research utilizing a digital tongue indicated that the dried salted mackerel exhibited the most pronounced aroma and salinity after thermal treatment. The concentration of aspartic acid and glutamic acid in the oil frying group (notably F64) was markedly elevated compared to the other two groups, and the quantity of flavor-contributing amino acids exhibited a positive correlation with the e-tongue analysis, suggesting that the salty and fresh flavors of the oil frying group surpassed those of the other two groups. LC-MS metabolite analysis was employed to examine the variations in free amino acid concentrations in the dried meat of salted mackerel subjected to various heating procedures. Subsequent to the sensory evaluation, the oil frying group (notably F64) surpassed the other two groups in all categories. This research can operate as a resource for enhancing the technology employed in the processing of heat-cured aquatic goods throughout production.

## Data Availability

The original contributions presented in this study are included in this article/supplementary material, further inquiries can be directed to the corresponding author.

## References

[1] Jiang C Liu Y Jin W Zhu K Miao X Dong X Effects of curing concentration and drying time on flavor and microorganisms in dry salted Spanish mackerel. *Food Chem X.* (2024) 21:101126. 10.1016/j.fochx.2024.101126 38292676 PMC10825358

[2] Wu S Yang J Dong H Liu Q Li X Zeng X Key aroma compounds of Chinese dry-cured Spanish mackerel (Scomberomorus niphonius) and their potential metabolic mechanisms. *Food Chem.* (2021) 342:128381. 10.1016/j.foodchem.2020.128381 33097327

[3] Hong H Luo Y Zhou Z Shen H. Effects of low concentration of salt and sucrose on the quality of bighead carp (Aristichthys nobilis) fillets stored at 4 °C. *Food Chem.* (2012) 133:102–7. 10.1016/j.foodchem.2012.01.002

[4] Wu H Wang Y Jiang Q Jiang X Feng Q Shi W. Changes in physicochemical properties and myofibrillar protein properties in grass carp salted by brining and injection. *Int J of Food Sci Tech.* (2021) 56:5674–87. 10.1111/ijfs.15108

[5] Mariutti L Bragagnolo N. Influence of salt on lipid oxidation in meat and seafood products: a review. *Food Res Int.* (2017) 94:90–100. 10.1016/j.foodres.2017.02.003 28290372

[6] Menis-Henrique M. Methodologies to advance the understanding of flavor chemistry. *Curr Opin Food Sci.* (2020) 33:131–5. 10.1016/j.cofs.2020.04.005

[7] Kosowska M Majcher M Fortuna T. Volatile compounds in meat and meat products. *Food Sci Technol.* (2017) 37:1–7. 10.1590/1678-457x.08416

[8] Zou Y Kang D Liu R Qi J Zhou G Zhang W. Effects of ultrasonic assisted cooking on the chemical profiles of taste and flavor of spiced beef. *Ultrasonics Sonochem.* (2018) 46:36–45. 10.1016/j.ultsonch.2018.04.005 29739511

[9] Yu X Li L Xue J Wang J Song G Zhang Y Effect of air-frying conditions on the quality attributes and lipidomic characteristics of surimi during processing. *Innov Food Sci Emerg Technol.* (2020) 60:102305. 10.1016/j.ifset.2020.102305

[10] Uran H Gokoglu N. Effects of cooking methods and temperatures on nutritional and quality characteristics of anchovy (Engraulis encrasicholus). *J Food Sci Technol.* (2014) 51:722–8. 10.1007/s13197-011-0551-5 24741166 PMC3982018

[11] Fan H Fan D Huang J Zhao J Yan B Ma S Cooking evaluation of crayfish (Procambarus clarkia) subjected to microwave and conduction heating: a visualized strategy to understand the heat-induced quality changes of food. *Innov Food Sci Emerg Technol.* (2020) 62:102368. 10.1016/j.ifset.2020.102368

[12] Wei H Wei Y Qiu X Yang S Chen F Ni H Comparison of potent odorants in raw and cooked mildly salted large yellow croaker using odor-active value calculation and omission test: understanding the role of cooking method. *Food Chem.* (2023) 402:134015. 10.1016/j.foodchem.2022.134015 36137382

[13] Chang L Lin S Zou B Zheng X Zhang S Tang Y. Effect of frying conditions on self-heating fried spanish mackerel quality attributes and flavor characteristics. *Foods.* (2021) 10:98. 10.3390/foods10010098 33466563 PMC7824904

[14] Esteki M Shahsavari Z Simal-Gandara J. Use of spectroscopic methods in combination with linear discriminant analysis for authentication of food products. *Food Control.* (2018) 91:100–12. 10.1016/j.foodcont.2018.03.031

[15] Wang S Chen H Sun B. Recent progress in food flavor analysis using gas chromatography–ion mobility spectrometry (GC–IMS). *Food Chem.* (2020) 315:126158. 10.1016/j.foodchem.2019.126158 32014672

[16] Liu X Locasale J. Metabolomics: a primer. *Trends Biochem Sci.* (2017) 42:274–84. 10.1016/j.tibs.2017.01.004 28196646 PMC5376220

[17] Huang J Kong X Chen Y Chen J. Assessment of flavor characteristics in snakehead (Ophiocephalus argus Cantor) surimi gels affected by atmospheric cold plasma treatment using GC-IMS. *Front Nutr.* (2023) 9:1086426. 10.3389/fnut.2022.1086426 36712526 PMC9875017

[18] López-Martínez M Toldrá F Mora L. Pork organs as a potential source of flavour-related substances. *Food Res Int.* (2023) 173:113468. 10.1016/j.foodres.2023.113468 37803790

[19] Merlo T Lorenzo J Saldaña E Patinho I Oliveira A Menegali B Relationship between volatile organic compounds, free amino acids, and sensory profile of smoked bacon. *Meat Sci.* (2021) 181:108596. 10.1016/j.meatsci.2021.108596 34118571

[20] Wang H Zhu Y Zhang J Wang X Shi W. Characteristic volatile compounds in different parts of grass carp by comprehensive two-dimensional gas chromatography/time-of-flight mass spectrometry. *Int J Food Properties.* (2020) 23:777–96. 10.1080/10942912.2020.1758715

[21] Luo X Xiao S Ruan Q Gao Q An Y Hu Y Differences in flavor characteristics of frozen surimi products reheated by microwave, water boiling, steaming, and frying. *Food Chem.* (2022) 372:131260. 10.1016/j.foodchem.2021.131260 34628122

[22] Fu Y Cao S Yang L Li Z. Flavor formation based on lipid in meat and meat products: a review. *J Food Biochem.* (2022) 46:14439. 10.1111/jfbc.14439 36183160

[23] Xie Q Xu B Xu Y Yao Z Zhu B Li X Effects of different thermal treatment temperatures on volatile flavour compounds of water-boiled salted duck after packaging. *LWT.* (2022) 154:112625. 10.1016/j.lwt.2021.112625

[24] Zhang C Shi R Mi S Chitrakar B Liu W Xu Z Effect of different thermal processing methods on flavor characteristics of Penaeus vannamei. *LWT.* (2024) 191:115652. 10.1016/j.lwt.2023.115652

[25] Rossini K Verdun S Cariou V Qannari E Fogliatto FS. PLS discriminant analysis applied to conventional sensory profiling data. *Food Quality Preference.* (2012) 23:18–24. 10.1016/j.foodqual.2011.01.005

[26] Zaukuu J Gillay Z Kovacs Z. Standardized extraction techniques for meat analysis with the electronic tongue: a case study of poultry and red meat adulteration. *Sensors.* (2021) 21:481. 10.3390/s21020481 33445458 PMC7827137

[27] Wang X Feng T Wang X Xia S Yu J Zhang X. Microwave heating and conduction heating pork belly: non-volatile compounds and their correlation with taste characteristics, heat transfer modes, and matrix microstructure. *Meat Sci.* (2022) 192:108899. 10.1016/j.meatsci.2022.108899 35797849

[28] Dashdorj D Amna T Hwang I. Influence of specific taste-active components on meat flavor as affected by intrinsic and extrinsic factors: an overview. *Eur Food Res Technol.* (2015) 241:157–71. 10.1007/s00217-015-2449-3

[29] Yin X Lv Y Wen R Wang Y Chen Q Kong B. Characterization of selected Harbin red sausages on the basis of their flavour profiles using HS-SPME-GC/MS combined with electronic nose and electronic tongue. *Meat Sci.* (2021) 172:108345. 10.1016/j.meatsci.2020.108345 33120175

[30] Troise A Buonanno M Fiore A Monti S Fogliano V. Evolution of protein bound Maillard reaction end-products and free Amadori compounds in low lactose milk in presence of fructosamine oxidase I. *Food Chem.* (2016) 212:722–9. 10.1016/j.foodchem.2016.06.037 27374589

[31] Yuan H Sun L Chen M Wang J. The comparison of the contents of sugar, amadori, and heyns compounds in fresh and black garlic. *J Food Sci.* (2016) 81:13365. 10.1111/1750-3841.13365 27300762

[32] Cai W Jiang P Liu Y Miao X Liu A. Distinct changes of taste quality and metabolite profile in different tomato varieties revealed by LC-MS metabolomics. *Food Chem.* (2024) 442:138456. 10.1016/j.foodchem.2024.138456 38271909

[33] Liao R Xia Q Zhou C Geng F Wang Y Sun Y LC-MS/MS-based metabolomics and sensory evaluation characterize metabolites and texture of normal and spoiled dry-cured hams. *Food Chem.* (2022) 371:131156. 10.1016/j.foodchem.2021.131156 34583183

[34] Wang S Jin N Jin L Xiao X Hu L Liu Z Response of tomato fruit quality depends on period of LED supplementary light. *Front Nutr.* (2022) 9:833723. 10.3389/fnut.2022.833723 35174200 PMC8841748

[35] Ma X Yang D Qiu W Mei J Xie J. Influence of multifrequency ultrasound-assisted freezing on the flavour attributes and myofibrillar protein characteristics of cultured large yellow croaker (Larimichthys crocea). *Front Nutr.* (2021) 8:779546. 10.3389/fnut.2021.779546 34977123 PMC8714677

[36] Ganguly A Pang L Duong V Lee A Schoniger H Varady E Molecular and cellular context-dependent role for Ir76b in detection of amino acid taste. *Cell Rep.* (2017) 18:737–50. 10.1016/j.celrep.2016.12.071 28099851 PMC5258133

[37] Sun X Zheng J Liu B Huang Z Chen F. Characteristics of the enzyme-induced release of bitter peptides from wheat gluten hydrolysates. *Front Nutr.* (2022) 9:1022257. 10.3389/fnut.2022.1022257 36267904 PMC9577220

[38] Wang W Zhou X Liu Y. Characterization and evaluation of umami taste: a review. *TrAC Trends Anal Chem.* (2020) 127:115876. 10.1016/j.trac.2020.115876

[39] Jin Y Xu M Jin Y Deng S Tao N Qiu W. Simultaneous detection and analysis of free amino acids and glutathione in different shrimp. *Foods.* (2022) 11:2599. 10.3390/foods11172599 36076785 PMC9455249

[40] Yang L Dai B Ayed C Liu Y. Comparing the metabolic profiles of raw and cooked pufferfish (Takifugu flavidus) meat by NMR assessment. *Food Chem.* (2019) 290:107–13. 10.1016/j.foodchem.2019.03.128 31000026

[41] George AB. *Fenaroli’s Handbook of Flavor Ingredients.* Boca Raton: CRC Press (2004).

